# Making universal digital access universal: lessons from COVID-19 in Singapore

**DOI:** 10.1007/s10209-022-00877-9

**Published:** 2022-04-15

**Authors:** Irene Y. H. Ng, Sun Sun Lim, Natalie Pang

**Affiliations:** 1 Department of Social Work, Social Service Research Centre, National University of Singapore, Singapore, Singapore; 2 Department of Humanities, Arts and Social Sciences, Singapore University of Technology and Design, Singapore, Singapore; 3 Department of Communications and New Media, National University of Singapore, Singapore, Singapore

**Keywords:** Universal, Digital access, Digital inclusion, COVID-19

## Abstract

Digital resources—which include devices, internet connection and digital literacy—have become basic needs. Thus with the global COVID-19 pandemic having accelerated digitalization, the urgency for universal digital inclusion has hastened. Otherwise, digital inequality will lead to social inequality and impede social mobility. Using Singapore as a case study, this article applies the insights learned from a participatory action research to recommend a policy framework for universal digital access, with practical humanistic steps towards full digital inclusion. Singapore is a digitally advanced nation with almost universal digital availability, yet when COVID-19 forced rapid digital adoption, gaps in access by vulnerable groups such as low-income households, elderly and migrant workers were found. From the learning points on gaps and measures taken by community groups, volunteers and policy-makers in our research, we recommend making access to all three digital resources automatic and affordable, with an undergirding principle to implement technology among the most digitally excluded first before national roll out. A public-community-corporate funding and partnership model is also proposed to sustain universal provision.

## Introduction

The global COVID-19 pandemic has accelerated digital adoption with lockdowns and work from home arrangements leading to a surge in online meetings, online education and online shopping. As safe distancing has stretched from weeks to months and even years, these digital activities will likely become a mainstay even after COVID-19 is brought to heel.

However, digitalisation has by no means been a straightforward process, with gaps emerging in many societies, including even in digitally connected countries such as Singapore. At the height of the pandemic, the urbanised city-state saw many low-income students without the necessary devices, internet connections or competencies to avail of online learning. Separately, elderly or migrant communities with limited English proficiency—the dominant digital and official working language—struggled to learn even basic mobile functions for communicating, banking and other online transactions.

Such challenges are sobering considering that Singapore has among the highest levels of digital coverage in the world. The 2019 IMDA Annual Survey on Infocomm Usage reports that 89% of resident households have access to a computer, and 98% have access to the internet [11]. Additionally, the consultancy firm Roland Berger’s Digital Inclusion Index 2020 ranks Singapore as the top among 82 countries for digital inclusiveness [25]. However, these statistics do not reveal the full picture on digital device ownership, usage duration or quality of online access. With the gaps spotlighted by COVID-19, it became clear that the level of access that allows vulnerable groups such as low-income households, elderly and migrant workers, to engage in the digital world to the extent required in a post-pandemic world is far from ideal even or especially in a country with high digital penetration.

Taking Singapore as a case study therefore, this paper discusses the notion of universal digital access, why universality as a concept has growing currency, and how it can be achieved. Singapore offers an apt illustration for several reasons. First, with its high digital penetration, lessons can be drawn from the digitalisation that Singapore had already embarked upon before the onset of COVID-19. Second, in light of the digital gaps triggered by Singapore’s COVID-19 lockdown, pointers can also be drawn for other economies with similarly high digital penetration. Third, the recommendations developed from our research offer new insights from which general principles can be applied to bridge digital gaps across a spectrum of countries.

The following section outlines the framework that this article is premised upon, that of universal digital access, and the related literature, and debates around making digital adoption more inclusive. The subsequent section then outlines the methods applied in this research, followed by a presentation of the findings on the location, extent and nature of digital gaps. The article rounds up with recommendations for bridging each gap and broader principles for consideration.

## Universality and access

The United Nations, in its report titled “Age of Digital Interdependence”, calls on governments, civil society, banks and the UN itself to work towards achieving full digital inclusion and digital equality [29]. Although the word “universal” was not used, the principles of full inclusion and equality evoke universalism which, broadly defined, refers to access for all. More concretely, the Journal *Universal Access in the Information Society (JUAIS)* defines universal access as “the conscious and systematic effort to proactively apply principles, methods and tools of universal design order to develop Information Society Technologies that are accessible and usable by all citizens, including the very young and the elderly and people with different types of disabilities, thus avoiding the need for a posteriori adaptations or specialized design” [30].

While the above definitions provide important frameworks for universal access, materializing universality requires greater specificity. Currently, in most countries around the world, access to digital resources from computing devices to internet access and skills, is far from universal. While prices of digital resources have greatly decreased from when computers were first invented, and their availability greatly expanded, affordability continues to elude vulnerable groups such as low-income households and persons with disabilities.

There are three reasons why digital access should be universal. First, in today’s highly digitalising world, even before COVID-19 emerged, digital access is indispensable for participating economically and socially, be it for work, education, recreation, or family life. It has thus become a need, and no longer a want as may have been true a decade or more ago. Thus, the starting premise of this article is that digital access should now be seen as equivalent to other necessities, such as those tracked by the United Nations’ human development index—clean water, electricity, and education. These are today considered public utilities where the state (and international development programmes in less developed countries) actively intervenes to ensure affordable access to all. In developed economies, these public utilities are taken for granted, where people expect access to clean water and electricity in residences and public spaces.

Second, while technology has become more pervasive, income inequality has also widened [9, 23]. From the above confluence of factors, digital inequality will become a source of social divides, exacerbating existing inequalities and impeding social mobility over time.

Third, COVID-19 has hastened digital transformation worldwide [21], making the first two reasons for universalising digital access all the more urgent. Notably, the pandemic’s adverse impact on physical and mental health necessitates that health services be readily forthcoming. With lockdowns being sporadically imposed around the world, digital health services such as telehealth and online counselling assume even greater importance. In such circumstances, those who are digitally-behind are consequently also excluded from a broad swathe of essential services, including health.

### Chasm between vision and implementation

While the principles for universal access are sound and encompassing, realising them is a significant undertaking. In social policy, universal provision is often juxtaposed with selective provision, i.e. targeted through means-and-needs-tests for only the neediest [1, 5]. Universalist policies are fiscally expensive, because they provide even for those who can afford to purchase the service privately. However, stringent assessments of selective services incur higher administrative costs and often lead to needy individuals lacking access.

In the case of digital access, our preceding discussion suggests that the social costs of digital inequality are likely to outweigh the fiscal cost of universal provision. However, even if a state articulates the goal of universal digital access, the outcome could still be unequal because full inclusion requires more than official proclamations and publicity campaigns. Singapore’s experience in this regard is revelatory in several ways. For example, in the country’s mainstream media, digital inclusion has regularly made news headlines for several years. On closer scrutiny though, it is unclear how effective these calls for digital inclusion actually have been. No less than the President of Singapore raised digital inclusion and illustrated it with the following examples: “digital inclusion of vulnerable groups in society will be the theme for next year's President's Challenge, using technology to engage with seniors who are isolated during the pandemic, or using data analytics to provide better service support for beneficiaries [7].”

Ostensibly, such motherhood statements are sensible, well-intentioned and suitably ambitious. However, the ground realities for realising them present significant obstacles. As we will discuss in subsequent sections, several vulnerable groups in Singapore encounter many practical hurdles in seeking digital connections. For instance, many elderly do not own smartphones, and those who do may only have old smartphones that lack updated operating systems to run the latest apps. Yet others may only be able to afford limited data plans such that their usage of particular apps will well exceed their plans and incur them steep charges they may be oblivious to because of their modest technological competencies. For digital access to be truly inclusive, policy makers must pay attention to such human factors as much as they apprise themselves of the latest technology tools.

The Information Media and Development Authority (IMDA) and GovTech are two government units leading the digital transformation efforts in Singapore. The next section on developments prior to COVID-19 will show that there was much deliberation over establishing principles to make Singapore’s digital roadmap inclusive. However, without knowledge of how the least digitally connected will interface with the myriad of programmes available, the principles remained as principles that failed to translate into universal adoption. Indeed, as Minister of State of the Ministry of Information and Communications Mr. Janil Puthucheary reflected in a conference held 1 year after COVID-19 first emerged, Singapore needs a more humanistic approach in digital transformation [24].

## Ground-up participatory action research

This research emerged from the authors’ personal involvement in various efforts pertaining to digital access. Two authors were involved in securing donated laptops for needy families, and one author had been involved in public education for boosting digital literacy. As we collaborated through publishing media commentaries and speaking at public webinars on the problems of digital exclusion, we were approached by individuals and civil society groups who volitionally shared their experiences and proposed solutions. This groundswell of views and interest spurred us to embark on further research to understand in-depth the issue of universal digital access in Singapore. Participatory action research was an appropriate approach because of our direct personal involvement in raising digital access for vulnerable groups.

To concretize our participatory action research method, we obtained institutional ethics clearance in October 2020. Our goal was to canvas as wide a range of relevant community views as possible on the issue of digital access and the consequences of its lack thereof. From October to December 2020, we conducted six work group and focus group discussions and two individual interviews with leads of two non-profit organisations providing donated laptops. We also exchanged many WhatsApp messages and e-mails with representatives of different social agencies or community groups involved in digital access initiatives to further probe the issue.

We subsequently presented our findings and proposals to relevant government officials whose reactions are also incorporated in this article. With COVID-19 restrictions on gatherings of large groups, all meetings were conducted online via platforms such as ZOOM, save for one site visit to Engineering Good, a non-profit that collected and refurbished donated laptops for donation to needy families and individuals.

## Singapore’s digital transformation roadmap

### Historical development

Since its independence in 1965, Singapore has enjoyed significant benefits from developing its technological infrastructure and applications. These gains strengthened the conviction to develop Singapore as a digitally advanced and competitive economy, as evidenced in several watershed IT plans and visions, starting in the 1980s.

In 1981, the National Computer Board (NCB) was established to spearhead the computerisation of work processes within the government bureaucracy, and also explore computerisation at a national level [17]. Subsequently, in 1986, the National IT Plan was launched with the goal of scaling up efforts to boost Singapore’s competitiveness via information technologies. This included several strategies to develop manpower and entrepreneurship in the IT industry, prepare people for an IT-driven society, improve information and communication infrastructure, and enhance IT applications across various economic sectors.

Besides driving economic growth, successive technology masterplans such as IT2000 (1991–2005) also sought to expand the use of IT to “enhance the potential of individuals”, “link communities locally and globally”, and “improve the quality of life…[through] increasing discretionary time, generating more opportunities and choices in leisure, kinship, work and civic spheres of life” [28]. To realise these goals, Singapore invested in the rapid development of comprehensive broadband infrastructure including the introduction of broadband internet into residences and the launch of the national free wireless internet network Wireless@SG. With the execution of each IT masterplan plan, Singapore’s progressive digitalisation was increasingly felt in all aspects of life including work, education, consumption, recreation, and government and banking services. Accordingly, individual and household adoption of computers, mobile phones and internet subscriptions also grew rapidly. For instance, between 2000 and 2010, computer usage among Singapore’s resident population increased from 49 to 71% while internet usage rose from 36 to 69% [11, 26].

The launch of the SmartNation initiative in 2014 further upped the ante on Singapore’s digitalisation drive. SmartNation distinguished itself from previous IT masterplans that focused on building and enhancing technological infrastructures by prioritising open data and analytics in various sectoral applications. For individuals and households, this meant that many public services and transactions that make up their daily routines, e.g. commuting, purchasing goods and services, paying taxes, have become more digitalised. Digitalisation was thus further cemented as part and parcel of everyday life in Singapore.

### Improving access for digitally excluded communities

Although household device ownership and infocomm usage were rising for the vast majority of households, low-income households bucked the trend. The NEU PC Plus programme was thus launched in 1999 to offer low-income households with school-going children and persons with disabilities access to personal computer ‘bundles’. This bundle includes access to broadband subscription as well as a personal computer at a subsidised rate. For low-income households with no school-going children, the Home Access Programme (HAP) was launched in 2014 to support these households with subsidized broadband connectivity and a tablet computer. A Social Innovations Grant was also initiated to encourage Voluntary Welfare Organisations to adopt IT solutions that directly benefit their clients.

These welcome steps towards greater digital inclusion were given a further boost with the launch of the Digital Readiness Blueprint in 2018 [22] that promulgated a set of Digital Services Standards for public agencies [8]. These standards require government websites to have intuitive design, easy navigation, and inclusive functionality, among other features. The Blueprint also signalled greater appreciation of the need for sharpened focus on the human factors of technology adoption: “Many people associate Singapore’s Smart Nation drive with hardware like devices and sensors. But [it] is really about making Singapore a great place to live, work, and play, powered by technology, for everyone” [22].

### Digital disruption and skills

As digitalization gained pace, the increasing need for a massive upgrade in digital competencies across the population led to various skills-focused initiatives including the Ministry of Education’s National Digital Literacy Programme launched in March 2020. This growing turn towards fortifying the functional and critical media literacy of Singaporeans across all age groups was also manifested in other government initiatives such as the Media Literacy Council’s Better Internet Campaign. Through such programmes, the national push on digital access had therefore broadened beyond hardware and infrastructure to encompass digital skills and competencies.

By the time COVID-19 landed on Singapore’s shores in March 2020, the country’s decades-long digital transformation had already borne fruit. To stall the pandemic’s spread, Singapore was forced to impose a nationwide lockdown that required all employees in all sectors save for essential services to work from home, and students across all levels, from pre-school through to university, to engage in online learning. For the most part, the country’s robust digital infrastructure could cope with the surge in online activity and except for a few teething pains, public and commercial services migrated online swiftly and seamlessly. However, cracks nevertheless emerged that highlighted inadequacies in digital access for various vulnerable communities whose experiences and challenges we next turn to.

## Critical gaps in digital access

Our discussion of the inadequacies in digital access will be organised according to three key components of digital access: computing devices, digital literacy, and internet access. In particular, we will probe into why these digital inadequacies occurred despite digital inclusion programmes that had been established well before COVID-19.

### Digital devices

The internet can be accessed through a diversity of devices, from desktop computers to laptops and tablets, as well as the ubiquitous smartphone. However, not all devices are made equal and clearly their respective affordances lend them better to different uses. For example, whereas the smartphone’s portability and multi-functionality make it a great tool for communication, entertainment and media production, its small screen size presents significant constraints. In particular, online learning platforms that involve the sharing of content by teachers, along with concurrent online interaction sections for students to ask and answer questions, and online avatars or thumbnails that represent students’ presence in virtual classroom cannot be optimally experienced via smartphones or even larger tablets. Instead, most online learning platforms are mainly designed for desktop or laptop computers.

#### Students need laptops

Globally therefore, one clear digital inequity spotlighted by the pandemic was that among students. As online learning was rolled out worldwide due to lockdowns and school closures, a slew of inequities in digital access were unleashed. While students from well-to-do families had ample resources to engage in online learning, reports emerged of low-income students sharing their parents’ mobile telephones or tapping on free Wi-Fi at fast-food restaurants to attend online classes and complete online assignments [3, 15, 16]. Similar accounts were noted in Singapore [18] that then begged the question as to why pre-existing digital support and inclusion schemes had failed to avert such problems.

As described previously, Singapore has two main means-tested programmes to enable device and internet ownership by low-income households. However, through our ground research, we identified the following reasons why these programmes were not more widely used. First, some people did not know of the existence of these digital support programmes. Awareness of available support was therefore a clear hindrance. Second, some households who needed the laptops did not meet certain criteria such as restrictions on each household being limited to one laptop. Prior to the pandemic, this restriction may have seemed reasonable. However, in families with more than one school-going child, and children having to attend online classes concurrently during school hours, such a device limit for needy households is a clear impediment. IMDA, the agency overseeing this programme, has since relaxed the criteria after COVID-19 by permitting a second laptop for households with three or more children of school-going age and waiving the community service requirement for additional subsidies [14]. As more online components are being systematically introduced into Singapore’s education system, such as online lectures, educational online games and homework submission portals, device support must be stepped up for needy families even during non-crisis periods. With the rising necessity of personal computers for each child’s learning, eligibility criteria for device support should be continuously reviewed.

Third, various factors deterred eligible households from applying for IMDA’s digital support programmes. Some were aware but did not apply, attributing their reticence to the stringent criteria and conditions. Others were put off by the challenges of accessing and completing the necessary paperwork. For example, NEU PC Plus is offered through schools, and thus needy students have to first be aware of the scheme, before taking the initiative to request the application forms from the school. The additional effort coupled with the stigma and embarrassment of applying could discourage the students who most need the programme from coming forward. Furthermore, the application process was also protracted, with each request having to be assessed through schools, MOE headquarters and IMDA. The speed with which households can get the laptops was undesirably slow.

Fourth, there was also lack of awareness of the importance of device ownership for school-going purposes. Previous research on Singapore has found that lower income households tend to be mobile-only or mobile-first, where due to their digital illiteracy and financial constraints, prefer to rely exclusively or primarily on their mobile phones for internet access [20]. This, therefore, poses a chicken-and-egg problem, where people who do not own computers do not see the need for them, and people who do not see the need for computers do not own them.

Thus, the factors we have just discussed strongly suggest that the means-and-needs-tested approach undergirding Singapore’s digital support programmes for low income families, up to when COVID-19 hit, has significant limitations. Fortunately, the Singapore government has not been oblivious to the heightened need for digital device support for school students. With e-learning suddenly becoming normative, MOE announced that it would bring forward to 2021 its plans to provide every secondary school student a laptop, seven years ahead of schedule. This, in effect, makes laptops a universal good for all secondary school students. However, practically, the programme is still not fully universal because parents are still expected to purchase the laptops for their children. Hence, access to subsidies is still subject to previously highlighted issues of whether parents are aware of and able to apply for the subsidies. The one-to-one laptop programme for secondary schools also remains short of the vision of universal device access for all. Younger students like those in primary schools also increasingly need computers for learning, as do adult learners given the growing impetus for continuing adult education and upskilling.

#### Adult learners need laptops too

Indeed, low-income working adults also require digital device support but the existing programmes do not cover laptops for adults’ online learning. One notable case uncovered by our ground research illustrates the necessity of consistent device ownership for adult learners. Devi (pseudonym) had registered herself for a government-subsidized course on Info-communications. However, Devi suddenly found herself without a laptop to attend her classes via WebEx when the primary school semester ended. She had been using a laptop on loan to her child who was attending primary school but it had to be returned when the semester had ended. In desperation, she asked her volunteer for help and received one within a week from the non-profit organisation Engineering Good.

Devi’s experience demonstrates the necessity of digital access for bridging income inequality and facilitating social mobility. Her example is all the starker against the backdrop of a concerted national drive to encourage skills upgrading through the country’s SkillsFuture programme [27]. With digital skills being of increasing importance in current and future workplaces, intermittent digital access experienced by less educated and lower resourced individuals prevents them from participating in skills upgrading courses. These are crucial to helping them improve their chances for higher paying jobs and better career prospects. Similarly, computers are helpful for those starting home businesses because they provide greater functionality than smartphones for interfacing with customers, managing transactions and enhancing the business’s online presence. These forms of work have proliferated during COVID-19, and low-income households should be better supported so that they can also avail of such opportunities.

#### Up-to-date devices required

For other vulnerable communities, the need for computing device support is less critical. However, they too can benefit from support in acquiring smartphones that can help them send and receive vital information, access government services, conduct financial transactions and keep in touch with their loved ones for well-being. Besides elderly users who might not have updated mobile telephones, migrant workers were another group highlighted to us. Migrant workers must now use smartphones to record and submit their temperatures online to their employers and to check in to different venues to facilitate contact tracing in case of COVID-19 infections. As many of them are breadwinners for their families back home, access to digital remittance services has also become vital. All of these interfaces only function well with smartphones that can support the latest mobile operating systems. Yet migrant workers’ low salaries make it difficult for them to acquire such devices and many settle for basic or even outdated phones.

In sum, different vulnerable communities require different forms of digital device support. Device assistance schemes must therefore be tailored accordingly for useful and sustainable results.

### Internet access

For the digitally excluded, their internet access is at best intermittent and sporadic, with bandwidth, data and time limitations being especially pronounced. They use public free Wi-Fi at malls, libraries, community centres, and other commercial establishments. While such public access is free and forthcoming in Singapore, it is available only during opening hours of the establishments. The physical settings in which to tap into them are also not always ideal for studying, attending online courses, conducting secure transactions or making calls of a personal or private nature.

As a modern urban society with a reported internet access rate of 98%, it came as a surprise when it was discovered during Singapore’s lockdown due to COVID-19 that some public rental apartments catering to low-income households lacked internet points, and that other low-income households did not have internet connections at home because they could not afford it. Donations of dongles were hastily made to these households for students’ online learning. However, these mobile options via smartphone subscriptions, pre-paid plans, or dongles have data ceilings and bandwidth limitations/speed throttling that do not permit concurrent access by several household members. Such a situation undermines the use of the internet for educational purposes since households with multiple children and working adults accessing the internet at the same time would have to decide for whom internet access should be prioritised. Students and adult learners may also find themselves unable to complete the necessary learning activities within the allocated time and data ceilings. Ultimately, for full digital inclusion, affordable home high-speed internet with Wi-Fi access is the goal to strive for.

Since then, a few non-profit organisations and groups have raised funds to work with telecommunication companies to set up Wi-Fi routers in communal areas in such apartment buildings. However, these voluntary efforts have limited scope to scale up or be replicated across other neighbourhoods because they require not only the expertise of telcos, but also donor funds and co-operation from the authorities. In the above-cited efforts, the Members of Parliament for residential areas where the Wi-Fi routers and corners are set up played a crucial role in permitting and supporting their installation.

Therefore, due to the large scale of investments involved, building the infrastructure for all to have internet access clearly requires active government intervention. This was plain to the organisations we studied, even as they blazed the trail to bring internet access to the most disadvantaged homes within their service boundaries.

### Digital literacy

Of the three components for universal digital access, digital literacy is easily the most complex even though its definitions are seemingly straightforward. Digital literacy has been defined as the ability to understand and use information in multiple formats from a diversity of sources when presented via computers and the Internet [6]. It has also been used to refer to the panoply of cognitive-thinking skills that consumers of digital information exercise [4]. Crucially, it has been observed that most new literacies, including digital literacy, share four assumptions: (a) they include new skills, strategies, dispositions, and social practices that are required by new ICTs; (b) they are integral to full participation in a global community; (c) they regularly evolve as their defining technologies transform; and (d) they are multifaceted and our comprehension of them is enhanced by diverse perspectives [19].

These definitions and assumptions help shed light on the intricate mix of challenges involved in boosting the digital literacy of the digitally-excluded. We have found crucial literacy gaps, from the most foundational of not knowing how to switch on laptops and connect online, to lack of understanding of online risks. At the same time, the technical support and efforts to deliver skills appeared to lack appreciation of these fundamental challenges, and could do with the incorporation of more diverse perspectives as technologies evolve. We next discuss these issues of foundational knowledge, cybersecurity and skills delivery in turn.

#### Foundational knowledge

Because digital literacy entails accessing and using information from a range of online sources via computing devices, prior ownership of devices and exposure to a wide spectrum of technology and online experiences are highly advantageous for acquiring digital skills. This is a classic illustration of the ‘rich get richer’ adage. Privileged families have the financial wherewithal to acquire digital devices and access online services, and their previous technology experience helps them build their digital literacy quickly, preparing them to adapt more readily to new technological innovations that enter the market. Furthermore, they are more likely to already be working in jobs that require the use of ICTs and are thus privy to training and organisational support that can fill any digital literacy gaps that emerge. Moving up the technology and digital skills ladder is therefore fairly smooth-sailing for them. In contrast, digitally-excluded families and individuals are held back by lacking devices or owning outdated ones, sporadic internet use and working in jobs where exposure to ICTs is very limited, if at all. The foundational knowledge on which their digital literacy can be built is thus very weak and demands support programmes that take this critical factor into account.

Our evidence from the ground clearly fleshes out this reality. We highlight two examples, one showing the lack of basic skills to even use a computer, and another illustrating the lack of capability in using online platforms. In the first example, the agencies we studied that provide donated laptops to needy students found that some parents’ digital knowledge was so lacking that they did not even know how to plug in and switch on a computer. Other knowledge gaps involved troubleshooting problems associated with uses of online platforms e.g. live conferencing, Zoom, Google Classroom, uploading or submitting documents, as well as hardware issues, e.g. microphone and camera problems, internet connection issues.

On the other hand, new laptops on loan from government agencies stoked different concerns. Low-income families did not dare to use the laptops for fear of damaging them and having to suffer financial consequences. Such reservations are understandable given that these families are already feeling the strain of financial pressure and do not wish to bear the responsibility of repairing a big-ticket item such as a laptop. Ignorance about technology was also noted among low-income elderly, many of whom owned smartphones but did not know how to use them beyond a few basic functions and living alone, they had no one to consult.

The second example starts with the fact that with Singapore’s push for e-government, many public services are now online. For instance, even before COVID-19, job-seekers at government appointed employment agencies had to make advance booking of appointments online. COVID-19 gave further impetus to more e-services, including online application for government financial assistance, tele-counselling and tele-medicine, all of which are likely to expand in the future. And yet, the people who are least able to use these online platforms may be the ones who need them most—low-educated and low-waged workers seeking employment assistance, or a non-ambulant elderly seeking a routine doctor’s consultation for a chronic illness. While some online services are accompanied by online guides, these are not always easy to comprehend, especially for the less digitally or language literate. Guides also tend to be in English, which many elderly and low-skilled migrant workers do not understand. Our ground insights further suggest that some online public service platforms are not user-friendly, rather cumbersome to navigate.

Thus, social service professionals and volunteers we interviewed have had to step in to help their clients book appointments at employment agencies and clinics, teach them how to complete and submit online forms and help them scan and upload requested documents. While these social service professionals could support in the interim, their primary role is to provide social services and not technical support. Yet the latter is what is increasingly needed in a digitalised society where people from all socioeconomic strata must access public services online.

#### Cybersecurity

With the growing use of online platforms for important daily transactions, many of which are financial in nature and necessitate the exchange of sensitive personal data, cybersecurity has become a crucial component of digital literacy. Since digitally excluded individuals have less knowledge of basic cyber hygiene and cyber safety when they start getting online, they are more vulnerable to threat actors (such as cyber criminals or nation states), who can exploit them through scams, fraud, identity or data theft. In our interconnected digital environment, these threat actors will target them as the weak link to infiltrate networks, because the threat actors can then use lateral movement across networks to reach more lucrative victims such as businesses or government officials. Separately, low-income households that received donated refurbished laptops are also susceptible to cybersecurity risks. These second hand devices are likely to run outdated software that would not have current security patches, making them vulnerable to malware or infiltration by threat actors. When these laptops are connected to school or business networks, the malware or threat actors would be able to enter those networks and carry out cyberattacks, cyber espionage, or cybercrime.

At the same time, digitally-excluded persons have less experience in accessing online content and would be more prone to misinformation, disinformation, and manipulation. For example, they may not recognise the typical tactics that purveyors of online falsehoods tend to exploit such as sensationalism, content doctoring and emotional manipulation. They also need to be warned about online falsehoods that carry extremist religious or racially chauvinistic content, which could otherwise contribute to self-radicalisation. Digital literacy training of the digitally-excluded must therefore teach them to critically evaluate online information, especially to identify online falsehoods that spread through social media and messaging tools. Indeed, since inequality in digital literacy can actually pose national security risks, there is a strong economic justification for a progressive approach to digital literacy training where more digital skills education must be prioritized for the least able among us. In a technologically-connected society, digital literacy has therefore become a public good that requires state intervention for the advancement of national security and redistributive justice.

#### Skills delivery

Technical support is an important tranche of building up people’s digital literacy over the long term, and will help to support a skills delivery method that is more sensitive to the particular learning obstacles that confront the digitally-excluded. In this regard, some new digital training initiatives recently undertaken can prove instructive. Notably, in the wake of COVID-19, Singapore launched the Digital Ambassadors initiative that employed more than 1000 full-time personnel to provide one-to-one digital support and guidance. These ambassadors largely targeted two groups recognised as needing additional support i.e. elderly (Seniors Go Digital) and stallholders from food centres (Hawkers Go Digital) [12, 13].

This initiative has distinct strengths. It identified clear target groups, stationed the ambassadors in convenient locations close to the communities they were serving, and offered personal one-to-one guidance that addressed literacy gaps directly rather than group-oriented training classes. Nevertheless, it had some shortcomings that suggest the need for further refinement. Notably, our research participants highlighted how non-ambulant elderly or those in institutional care such as nursing homes are unable to visit the Digital Ambassadors at their stations. More importantly, our interviewees opined that if usage of digital devices is not part of their lives, the elderly might not approach the Digital Ambassadors for help, leading to the circular problem where elderly who most need to know how to use digital devices do not request assistance. Raising awareness on the benefits of digital devices and services is therefore an important first step in boosting digital skills.

Another useful takeaway is the importance of sustained training and guidance for the digitally-excluded in particular. This was the experience of a community organization helping the elderly learn to use mobile telephones and tablets for accessing government and other services. After running one-off workshops and finding that their elderly participants did not remember what was taught, they instead mobilized volunteers who mentored participants either one-on-one or in small groups for a period of time. Indeed, literacy in anything cannot be achieved through a one-off session but requires continued use and practice, and this in turn requires some level of confidence for the individual to keep using the new technology. Fundamentally however, because sustained and constant practice is critical to strengthening digital skills, device ownership and internet access are critical to the success of any digital training programme. As we will recommend in the conclusion, training and support for devices and internet access must go hand-in-hand.

## A framework for universal digital access

With the COVID-19 lockdown presenting the ideal stress-test for digital inclusiveness in Singapore, we can retrospectively assess how effective the range of measures and responses were in plugging the gaps. No doubt, Singapore’s early head start in laying the infrastructure for digitalization demonstrates the necessity for first investing in infrastructure that enables digital inclusion. However, despite the strong nation-wide initiatives in Singapore, uneven distribution of infrastructural support was found, e.g. the lack of internet connection points in older rental apartments and unaffordability to vulnerable populations of devices necessary for health declaration and contact tracing. In fact, the intense digitalisation being witnessed in countries like Singapore makes the exclusion of vulnerable groups more unacceptable because digital access has become imperative for essential services, educational opportunities, skills upgrading, professional advancement and social mobility. If low-income families and individuals are not provided with support for digital access, they will fall further behind from their inability to tap all of these opportunities, further widening existing social inequalities.

**Fig. 1:**
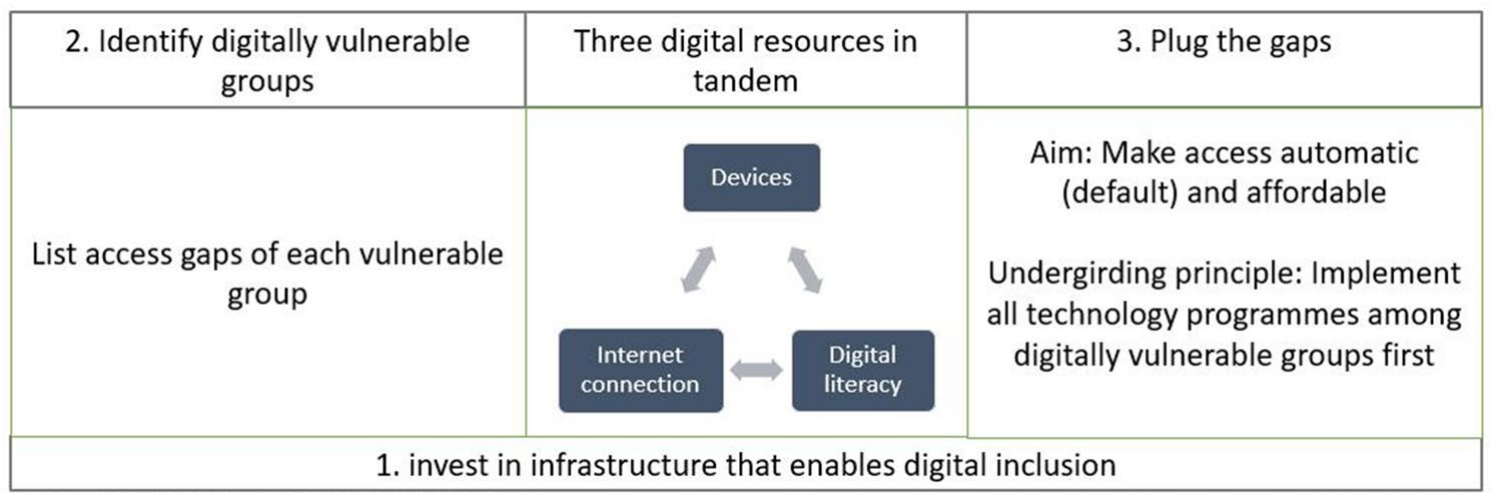
A policy framework for universal digital access

We therefore propose universal digital access as the modus operandi where digital resources are automatically and affordably provided as public utilities. This includes devices, internet connection, and digital literacy, all of which need to be universally available. Based on the insights we have garnered from our research, we posit here a policy framework for universal access to these three digital resources (Fig. 1). First, state investment in digital infrastructure is needed as the foundation on which to build universal access. Users can refer to the national blueprints and frameworks discussed earlier for the principles that can be adapted to different contexts. Second, the vulnerable groups in society are to be identified, and gaps in their access to each of the three digital resources articulated. In this paper, we have given examples of groups which include low-income children and adults, elderly and migrant communities.

Third, ways to plug the gaps by making the resources more automatically and affordably available are to be explored. For this, we list below goals and principles that digitalising societies should aim for, starting with an undergirding principle to implement all new technology programmes for the most excluded users first prior to national roll out. This will entail programme curators to seek out the inputs and responses of the most disadvantaged groups, and practitioners who work with them. It will also help iron out kinks before initiatives have already been made available to the larger population, by which time it might be too late to reverse gear and resultantly push the most vulnerable behind. Problems of low take-up of programmes or insufficient reach can be averted with an inclusion by design approach.

While different national contexts might germinate different aims and goals, we suggest the following aims for each digital resource.

For digital devices:

- Aim for one device per person; one laptop per learner;
- Incorporate laptops into computations of necessities, e.g. in training course fees, school’s book lists and financial assistance schemes;
- Tap on expertise and networks of existing tech charities to provide the technical manpower for tele services and laptop replacement in case of device spoilage.

For internet connection:

- Ensure internet connection in every home;
- Implement new technology (e.g. 5G) in disadvantaged communities before national roll out.

For digital skills:

- Invest in offering online government and other essential services such as financial, health and education in non-English speakers’ native languages, and secure but easy-to-use interfaces;
- Build digital education into school curricula, prioritizing schools with larger proportions of students from low-income households, and reaching parents in addition to students;
- Adopt a mentoring approach to digital education, where the mentor coaches the learner for a period of time.

## Whither government-community-corporate partnerships?

Achieving universal digital access requires state leadership, in partnership with community groups and corporate organizations. Over the crisis period, Singapore witnessed a commendable combination of government support measures, community initiatives as well as corporate programmes to offer digital access support to marginalised communities. For families with school children, the government provided laptops and mobile data dongles for loan but in view of the time pressure, there were still shortfalls. Voluntary community efforts stepped in with donated refurbished laptops. However, the volunteers we spoke to were clear that while they were able to mobilize tremendous networks and resources, their efforts were at best short-term ‘band aid’ solutions with inherent deficiencies. Notably, refurbished laptops tend to have shorter lifespans and are often not compatible with new software versions that are required to run various online learning applications. Some of the donated laptops also malfunctioned and required replacement in less than a year. The agencies we spoke to have started providing workshops and skills training, teaching recipients to repair their own laptops, etc. This affords greater competence and confidence in using digital resources. Still, each agency’s effort reaches only a limited group of people.

Thus, if full digital inclusion is to materialize, these inherent issues with voluntary efforts ultimately require state intervention and coordination for national digital inclusion for the long-term. This is especially so if universal access means digital resources for every person. A balance also needs to be struck between state intervention and leaving room for voluntary efforts. State leadership is clearly necessary, though the involvement of social service professionals, volunteers and vulnerable users themselves is also clearly a must not only for implementation of universal access, but also for the design of the policies and programmes.

In addition, corporate partnership is key, especially in terms of funding and technical services. The investments needed to achieve universal digital access will undoubtedly be greater than present targeted efforts. In countries such as Singapore, which have already committed heavily to digital transformation, some existing resources could be rechannelled towards inclusion. But in the long term, greater financial investments will be required. Therefore, a more sustainable funding model for national digital inclusion could be one that includes a public–private funding mix. At one level, corporate support can be sought for specific programmes based on an ability-to-pay principle, e.g. to fund Wi-Fi access for public rental blocks and low-income households through adopt-a-block or adopt-a-household programmes.

More comprehensively, big technology companies could be taxed. Huang and Cox [10] documented and analysed Taiwan’s model of funding and providing digital resources to low-income and homeless persons. They suggested “a social entrepreneurial system”, where telecommunications companies are required to contribute to the Telecommunications Universal Services (TUS) Fund by market share. This model can be adapted and expanded to big technology companies. As these companies have been able to leverage vast troves of user data for profit generation [2, 31], requiring them to contribute directly to full digital inclusion is not unreasonable and would be a form of wealth redistribution.

## Conclusion

The practical framework we have suggested offers a more humanistic approach to digital inclusion [24]. This implies that the launch of universal digital access cannot be tasked only to units with technical expertise, but more importantly in partnership with units that have intimate social knowledge about digitally-excluded communities, and business knowledge to involve businesses in corporate digital responsibility. Therefore, making universal digital access universal requires a holistic and deliberative collaboration between community organizations, corporations and government units in charge of technology as well as social welfare. To sustain government redistribution to equalize digital access, some form of Big Tech companies’ taxation could be imposed. It is time to rebalance access, because in a world transforming through technology, digital access is a need for everyday life and a critical source of social mobility.

## Data Availability

Not applicable.
